# Molecular detection and identification of *Leishmania* DNA and blood meal analysis in *Phlebotomus (Larroussius)* species

**DOI:** 10.1371/journal.pntd.0008077

**Published:** 2020-03-26

**Authors:** Latifa Remadi, Najla Chargui, Maribel Jiménez, Ricardo Molina, Najoua Haouas, Estela González, Raja Chaabane-Banaouas, Eya Ben Salah, Mohsen Haddaji, Yassine Chaabouni, Hamouda Babba

**Affiliations:** 1 University of Monastir, Faculty of Pharmacy, Laboratory of Medical and Molecular Parasitology-Mycology LP3M (code LR12ES08), Department of Clinical Biology B, Tunisia; 2 Laboratory of Medical Entomology, National Center for Microbiology, Instituto de Salud Carlos III, Majadahonda, Madrid, Spain; 3 Department of Clinical Laboratory Sciences, College of Applied Medical Sciences, University of Hail, Hail, Kingdom of Saudi Arabia; 4 Regional Commissariat for Agricultural Development of Kairouan, Tunisia; 5 Department of Biochemistry, CHU Ibn Jazzar, Kairouan, Tunisia; Faculty of Science, Ain Shams University (ASU), EGYPT

## Abstract

**Background:**

*Phlebotomus* (*Larroussius*) *perniciosus* and *Canis familiaris* are respectively the only confirmed vector and reservoir for the transmission of *Leishmania* (*L*.) *infantum* MON-1 in Tunisia. However, the vector and reservoir hosts of the two other zymodemes, MON-24 and MON-80, are still unknown. The aim of this study was to analyze the *L*. *infantum* life cycle in a Tunisian leishmaniasis focus. For this purpose, we have focused on: i) the detection, quantification and identification of *Leishmania* among this sand fly population, and ii) the analysis of the blood meal preferences of *Larroussius* (*Lar*.) *sub*genus sand flies to identify the potential reservoirs.

**Methodology and findings:**

A total of 3,831 sand flies were collected in seven locations from the center of Tunisia affected by human visceral leishmaniasis. The collected sand flies belonged to two genus *Phlebotomus* (*Ph*.) (five species) and *Sergentomyia* (four species). From the collected 1,029 *Lar*. *sub*genus female sand flies, 8.26% was positive to *Leishmania* by ITS1 nested PCR. Three *Leishmania* spp. were identified: *L*. *infantum* 28% (24/85), *L*. *killicki* 13% (11/85), and *L*. *major* 22% (19/85). To identify the blood meal sources in *Ph*. *Lar*. *sub*genus sand flies, engorged females were analyzed by PCR-sequencing targeting the vertebrate cytochrome *b* gene. Among the 177 analyzed blood-fed females, 169 samples were positive. Sequencing results showed seven blood sources: cattle, human, sheep, chicken, goat, donkey, and turkey. In addition, mixed blood meals were detected in twelve cases. *Leishmania* DNA was found in 21 engorged females, with a wide range of blood meal sources: cattle, chicken, goat, chicken/cattle, chicken/sheep, chicken/turkey and human/cattle. The parasite load was quantified in fed and unfed infected sand flies using a real time PCR targeting kinetoplast DNA. The average parasite load was 1,174 parasites/reaction and 90 parasites/reaction in unfed and fed flies, respectively.

**Conclusion:**

Our results support the role of *Ph*. *longicuspis*, *Ph*. *perfiliewi*, and *Ph*. *perniciosus* in *L*. *infantum* transmission. Furthermore, these species could be involved in *L*. *major* and *L*. *killicki* life cycles. The combination of the parasite detection and the blood meal analysis in this study highlights the incrimination of the identified vertebrate in *Leishmania* transmission. In addition, we quantify for the first time the parasite load in naturally infected sand flies caught in Tunisia. These findings are relevant for a better understanding of *L*. *infantum* transmission cycle in the country. Further investigations and control measures are needed to manage *L*. *infantum* transmission and its spreading.

## Author summary

*Leishmania* (*L*.) *infantum* is responsible for both visceral leishmaniasis and sporadic cutaneous leishmaniasis in Tunisia. The isoenzymatic typing of this taxon revealed three zymodemes and only one (*L*. *infantum* MON-1) presents the transmission cycle elucidated. In this study, we conducted an entomological survey using CDC light traps in central Tunisia wherein the three zymodemes of *L*. *infantum* coexist, to investigate the presence of *L*. *infantum*, to quantify the parasite load, and to analyze the blood meal sources in infected sand flies belonging to *Larroussius* (*Lar*.) *sub*genus. Our results demonstrate the role of *Ph*. *Lar*. species in *L*. *infantum* transmission and their potential role in *L*. *major* and *L*. *killicki* life cycles. The high parasite load observed in *Ph*. *perfiliewi* underline its incrimination in *L*. *infantum* transmission. Also, blood meal analysis showed that *Lar*. *sub*genus sand flies fed on cattle, goat, sheep, chicken, and human. Thus, in the light of the present results, further studies should be performed for a better understanding of *L*. *infantum* transmission cycles.

## Introduction

Leishmaniases are vector-borne diseases caused by *Leishmania* (*L*.) protozoan parasites and are transmitted to humans by the bite of infected female sand flies. Leishmaniases are widespread across 98 countries and 3 territories on 5 continents, with more than 58,000 visceral leishmaniasis cases (VL) and 220,000 cutaneous leishmaniasis cases (CL) per year [1]. In the Mediterranean basin, these two clinico-epidemiological forms of leishmaniasis coexist. Tunisia is endemic for leishmaniases, presenting a higher prevalence for CL compared to VL [1]. Indeed, CL is characterized by a large spectrum of clinical forms and caused by three *Leishmania* species: *L*. *major*, *L*. *infantum*, and *L*. *killicki* (synonymous *L*. *tropica*) [2, 3]. *Leishmania major* has a zoonotic transmission cycle with *Phlebotomus* (*Ph*.) *papatasi* as vector. *Psammomys obesus*, *Meriones shawi*, and *Meriones libycus* are the described reservoirs for this parasite, while *Mustela nivalis*, *Paraechinus aethiopicus*, and *Atelerix algirus* are potential reservoirs [4–7]. *Leishmania killicki*, the agent of the chronic CL in Tunisia, shows also a zoonotic life cycle with *Ph*. *sergenti* and *Ctenodactylus gundii* as potential vector and reservoir, respectively [8–11]. *Leishmania infantum* is responsible for both sporadic CL and VL. The isoenzymatic typing of this species has revealed three zymodemes, MON-1, MON-24, and MON-80 [3]. Until date, only the life cycles of CL and VL caused by *L*. *infantum* zymodeme MON-1 have been elucidated. Thus, *Ph*. *perniciosus* has been reported to be the vector and the domestic dog has been described as reservoir [12, 13]. Nevertheless, the vector and the reservoir hosts of the other SCL and VL causative zymodemes are still unidentified [14]. Previous studies have described *L*. *infantum* zymodeme MON-24 and MON-80 isolated in some dogs (in Algeria and Tunisia). However, we have to highlight that no one of these reports has fulfilled all the criteria to conclude to the reservoir role of the suspected animal. These criteria include: i) high animal population density, ii) spatial proximity to transmission cycles and humans and iii) high prevalence of infection without acute disease signs and with parasite forms present in the skin or bloodstream [15, 16]. Since then, several epidemiological and entomological surveys have been conducted to identify the vectors and the reservoirs of these undefined cycles.

Studies carried out in Tunisia have reported sand flies specimens of *Ph*. *langeroni*, *Ph*. *longicuspis*, *Ph*. *perfiliewi*, *Ph*. *perniciosus*, *Ph*. *papatasi*, and *Sergentomyia* (*Ser*.) *minuta* infected with *L*. *infantum* DNA using conventional PCR [17–19]. At present, many studies have been performed for *Leishmania* detection and quantification by using real time PCR (qPCR) assay. Thus, parasite loads help to understand the persistence and the development of *Leishmania* in sand fly midgut since a high parasite load is correlated to strong evidence of *Leishmania* transmission [20, 21]. Indeed, qPCR for *Leishmania* detection and quantification was used for *Ph*. *duboscqi*, *Ph*. *sergenti* in Iran, *Ph*. *papatasi*, *Ph*. *alexandri* in Iraq, *Ph*. *perfiliewi*, *Ph*. *perniciosus*, *Ph*. *neglectus*, in Italy, *Lutzomyia longipalpis*, *Lutzomyia migonei* in Brazil, and *Ph*. *perniciosus* in Spain [20, 22–28]. In Tunisia, Benabid et al., have performed qPCR targeting the kinetoplast DNA (kDNA) to assess *Leishmania* infection in sand flies and only *Ph*. *perniciosus* species has been found infected by *L*. *infantum* [29]. However, parasite loads have not been determined, and their estimation would bring additional information in vector competence studies.

On the other hand, feeding and host preference are key factors in determining the suspected reservoirs. In this sense, there are several works focused on the study of the blood meal in engorged females of *Larroussius sub*genus. An entomological survey conducted in the Center East of Tunisia reported two blood meal sources: cattle and horse in *Ph*. *perniciosus* and *Ph*. *longicuspis*, respectively [19]. Moreover, cattle, sheep, and wild rabbits were identified in engorged *Ph*. *perniciosus* collected in the CL focus situated in the center of the country [30]. However, the conducted studies were limited to a small number of engorged sand flies belonging to *Lar*. *sub*genus. So it would be interesting to investigate in *L*. *infantum* foci to analyze a bigger number of sand flies and identify the potential reservoirs.

In the present study, an epidemiological investigation of leishmaniasis caused by *L*. *infantum* was conducted in an endemic area of both human CL and VL aiming to: i) identify *Leishmania* in sand flies belonging to *Lar*. *sub*genus, ii) quantify the parasite loads in infected sand flies using qPCR and iii) assess the blood meal feeding behaviors of sand flies belonging to *Lar*. *sub*genus to identify the vector feeding preferences and potential mammalian reservoirs.

## Material and methods

### Sand fly collection

The study was carried out in the governorate of Kairouan, center of Tunisia (between 35°40’ N and 10° 05’ E). A semi-arid climatic conditions weather conditions characterize this region. The mean temperatures for the entire region rise between 9°C and 22°C. During the summer, the temperature typically rises as high as 40°C [31]. The area is composed of hills and plains with the presence of different types of cultivation and farming irrigation, making it convenient for sand flies population and peridomestic animal spreading. Kairouan is known as a heterogeneous focus of both CL and VL. Indeed, since 1982, the annual incidence of cutaneous and visceral leishmaniasis in Kairouan region was about 1044 and 45 cases respectively [32, 33]. Besides, we considered that this region is the more suitable focus to study *L*. *infantum* life cycle since three zymodemes of *L*. *infantum* (MON-1, MON-24, and MON-80) were isolated and identified. Indeed, the isoenzymatic analysis of isolated strains causing VL in this region has shown that *L*. *infantum* MON-1 was the most identified zymodeme (61.11%) followed by MON-24 (33.33%) and MON-80 (5.55%) [3].

Our study was conducted for three years (2014–2016) in the vicinity of human VL cases houses. The collection was carried out one day per week, from July to October during the activity peak period of the vector. In 2014, seven sites were analyzed. However, in 2015 and 2016 only four sites were re-analyzed, corresponding to those locations where infected *Larroussius* species were abundant (Table 1). CDC miniature light traps were placed outdoors, close to houses and animal shelters. Five traps were placed in each site on the sunset and removed before the sunrise of the next day. Before collection, all landowners were contacted, and all traps were set up with their permission.

**Table 1:** Geographical and ecological characteristics of sand flies collection sites.

Region code	GPS reference	Year of collection	Total number of traps	Animals at proximity
**Region A**	35°23’49.6"N 10°03’00.1"E	**2014**	**5**	**chicken, turkey, goat, sheep, dog, cat, horse, donkey, rabbit**
		**2015**	**5**	
		**2016**	**5**	
**Region B**	35° 56′ N, 10° 01′ E	**2014**	**5**	**dog, horse, donkey, horse, sheep, cat, chicken, goat**
		**2015**	**5**	
		**2016**	**5**	
**Region C**	35°24’11.8"N 9°57’58.3"E	**2014**	**5**	**cattle, goat, sheep, chicken, dog, cat, turkey, horse, donkey**
		**2015**	**5**	
		**2016**	**5**	
**Region D**	35° 37′ 07″ N, 9° 55′ 34″ E	**2014**	**5**	**sheep, goat, chicken, horse, dog, cat, turkey, rabbit**
		**2015**	**5**	
		**2016**	**5**	
**Region E**	35° 50′ N, 9° 35′ E	**2014**	**5**	**chicken, sheep, goat, cat, dog**
**Region F**	35° 21′ N, 9° 49′ E	**2014**	**5**	**chicken, sheep, goat, cat, dog**
**Region G**	35° 38′ N, 9° 40′ E	**2014**	**5**	**chicken, sheep, goat, donkey, cattle, cat, dog**

### Sand fly dissection and identification

After collection, traps were transferred directly to the laboratory where sand flies were sorted using a mouth aspirator. Individual specimens were transferred to a glass slide, the head and the three posterior segments of the abdomen were dissected and mounted in Hoyer mounting medium as described before for taxonomic identification [34]. Morphological identification of phlebotomine species was carried out according to the differential characters described in the identification keys [35–37]. Remaining parts of the abdomen were stored in ethanol 70% at -20°C in a sterile microtube until DNA extraction. Female sand flies were classified into three categories according to their abdomen state (engorged, unfed, and gravid). Only females belonging to *Lar*. *sub*genus were included in the molecular study.

### PCR detection and typing of the parasite

#### PCR detection of the parasite

DNA was extracted from sand flies belonging to *Lar*. *sub*genus using DNeasy Blood & Tissue extraction Kits (QIAGEN, Hilden, Germany) according to the manufacturer’s instructions. DNA samples were eluted in 100 μl of Tris EDTA buffer and stored at -20°C. DNA was quantified and its purity analysed using a Nanodrop 2000c spectrophotometer (Thermo Scientific). Screening for *Leishmania* infection was performed via PCR amplification of the 18S ribosomal RNA using primers previously described [38].

#### PCR typing of the parasite

In order to identify *Leishmania* species, nested PCR amplification of the ribosomal internal transcribed spacer 1 (ITS1) region was used as previously described with few modifications [39]. Two PCR reactions in separate tubes were conducted. In the first PCR, the ITS1 locus of the *Leishmania* ribosomal DNA was amplified using the following primers: LITSR-D, 5’CTGGATCATTTTCCGATG 3’, and L5.8S-R, 5’TGATACCACTTATCGCACTT 3’. The reaction was performed in a final volume of 50 μl containing 1× PCR buffer, 1.5 mM MgCl_2_, 200 μM deoxynucleotides, 0.5 pM of each primer, and 2U Taq DNA polymerase and 60 ng of extracted DNA. The amplification protocol is as follow: an initial denaturation at 94°C for 15 min followed by 34 cycles at 95°C for 20s, 53°C for 30s, 72 °C for 1 min and a final extension at 72 °C for 10 min. Then, 25 μl of the first PCR products were diluted in 1 ml of PCR-grade H_2_O, and 10 μl of these dilutions were used as template for the second PCR using the primers SAC-D, 5’CATTTTCCgATgATTACACC3’, and VAN2-R, 5’ gCgACACgTTATgTgAgCCg3’. Amplification was performed following the same conditions of the first PCR. Two negative controls for sample DNAs and PCR reagents were used to assess contamination in nested PCR. Positive samples to the nested PCR were digested with *HaeIII* enzyme, and PCR digestion products were visualized through a 12% polyacrylamide gel stained with ethidium bromide solution. Specific band patterns were observed under UV light exposure. To confirm PCR-RFLP results, some samples were verified by sequencing.

### Blood meal analysis

The approach used is based on a PCR amplification of a 359 bp fragment of the vertebrate cytochrome *b* (*cyt b*) gene with universal primers cyto 1 and cyto 2 according to the protocol of Jiménez et *al*. [34]. PCR was performed with 50 ng of extracted DNA in a final volume of 25 μl. Amplicons were analyzed by electrophoresis in 1.5% agarose gel. For negative samples, a second PCR was performed using a pair of degenerated primers: cyt bb1 (5’-CCATCMAACATYTCADCATGATGAAA-3’) and cyt bb2 (5’-GCHCCTCAGAATGAYATT TGKCCTCA-3’) according to the protocol described by González et *al*. [40]. The PCR products were purified and sequenced.

### *Leishmania* quantification in infected sand flies

Parasite loads were quantified in seventeen infected sand flies (those we had enough volume of DNA) by using a qPCR. Kinetoplast minicircle primers JW11 (5’-CCTATTTTACACCAACCCCCAGT-3’) and JW12 (5’-GGG TAGGGGCGTTCTGCGAAA-3’) were used according to the protocol described by González et *al*. [20, 41]. Two negative controls (non-template control (NTC) and negative control (NC) from reared sand flies) and two positive controls (10^3^ and 10^6^ promastigotes dilutions) were included in each PCR reaction. After amplification with the Corbett Rotor-Gene 6000 real-time PCR System (Qiagen), threshold cycle (Ct) values were calculated by Rotor-Gene Series Software version 1.7.

### Sequencing and analysis methodologies

The PCR products were purified using a QIAquick Gel Extraction Kit (QIAGEN), and DNA concentration was quantified for each positive sample. The PCR products were sequenced with ABI PRISM 3730XL DNA Analyzer (Applied Biosystems, EEUU). Sequences were edited using the BioEdit v7.0.0.1. program. Nucleotide sequences obtained were analyzed with the DNASTAR (Lasergen v7.1, Madison, WI, USA). Homologies with the available sequences data in GenBank were carried out with the Blastn software (http://www.ncbi.nlm.nih.gov/BLAST).

### Phylogenetic analysis

Partial ITS1 sequences obtained with nested PCR of infected sand flies and three *Leishmania* reference strains were analyzed. Reference strains were obtained either from “Centre National de Référence des leishmanioses, Montpellier France” (*L*. *major* code MHOM/MA/81/LEM265) or isolated in our laboratory for which the isoenzymatic identification was confirmed in the above-cited reference center (*L*. *killicki* code MHOM/TN/2005/LC05 and *L*. *infantum* code MHOM/TN/2003/23S). These strains were chosen to cover the three *Leishmania* species existing in Tunisia foci (*L*. *major*, *L*. *killicki*, and *L*. *infantum*). Besides, a partial ITS1 sequence of *L*. *siamensis* was recovered from Gene Bank data base (JX898938.1) and used as an outgroup to anchor the tree. Phylogenetic analysis was performed with MEGA X software version 10.0.4 using the Maximum Likelihood method and Kimura 2-parameter models. The tree topology was supported by 1000 bootstrap replicates.

### Statistical analysis

Statistical analysis was performed with GraphPad Prism 8 software. The non parametric statistical Kruskal-Wallis method was used to calculate significant differences between sand flies species distribution and parasite infection. Fisher’s exact test was used to compare parasite loads. Results were considered statistically significant when *p*-values were less than 0.05.

### Accession numbers

Accession numbers of sequences used in this study: MK474646, MK474647, MK474648, MK474649, MK474650, MK474651, MK474652, MK474653, MK474640, MK474641, MK474642, MK463621, MK463622, MK463623, MK463624, MK463625, MK463626, MK463627, MK463628, MK463629. Accession numbers of sequences discussed in this study: MG980399, KP691596, KY963132.

## Results

### Phlebotomine sand fly collection

Sand flies were collected at seven different locations where at least one case of human VL has been recorded before. A total of 3,831 sand flies were caught (2,049 males and 1,782 females), being the sex ratio 1.14. Details of the collected specimens are shown in Table 2. The study of phlebotomine sand fly fauna in these regions revealed the presence of nine species belonging to two genera, *Sergentomyia* (four species) and *Phlebotomus* (five species). The most abundant species was *Ph*. *papatasi* (N = 1,253; 32.70%) followed by *Ph*. *perniciosus* (N = 1,146; 29.91%), *Ph*. *perfiliewi* (N = 710; 18.53%), *Ph*. *longicuspis* (N = 426; 18.53%), and a few specimens of *Ph*. *sergenti* (N = 21, 0.54%). In addition, four species of sand flies belonging to *Sergentomyia* genus were identified: *Ser*. *fallax* (N = 124; 3.23%), *Ser*. *minuta* (N = 76; 1.98%), *Ser*. *dreyfussi* (N = 59; 1.54%), and *Ser*. *clydei* (N = 1; 0.02%). Species belonging to *Lar*. *sub*genus were the most abundant (N = 2,297; 59.95%). Statistical analysis through the non-parametric statistical Kruskal-Wallis method showed significant differences between the number of sand fly species in each site with *p*-value < 0.0001. Concerning the seven regions studied, only four sites (A, B, C, and D) were compared for sand fly abundance during the three years of collection using the Chi-square (Fisher’s exact) test. The difference was statistically significant (X^2^ = 734.7; *p*-value < 0.0001). The highest number of sand flies was found in region C (N = 1,547; 40.38%) followed by region A (N = 1,122; 29.28%), region B (N = 653; 17.04%), and region D (N = 300; 7.83%).

**Table 2:** Species identification of collected sand flies.

Genus		*Sergentomya*								*Phlebotomus*												Total		*P*
Species		*Ser*. *dreyfussi*		*Ser*. *minuta*		*Ser*. *fallax*		*Ser*. *clydei*		*Ph*. *sergenti*		*Ph*. *papatasi*		*Ph*. *perniciosus*		*Ph*. *perfiliewi*		*Ph*. *longicuspis*		*Lar*. spp.		Nbre	%	
Sex		F	M	F	M	F	M	F	M	F	M	F	M	F	M	F	M	F	M	F	M			
Collection site																								
**Region A**	**2014**	**2**	**7**	**6**	**13**	**10**	**19**	**0**	**0**	**1**	**5**	**76**	**100**	**62**	**164**	**2**	**103**	**53**	**11**	**1**	**0**	**635**	**29.28**	<0,0001* <0,0001**
	**2015**	**3**	**0**	**3**	**6**	**6**	**6**	**0**	**0**	**0**	**0**	**24**	**45**	**36**	**100**	**37**	**52**	**34**	**55**	**3**	**0**	**410**		
	**2016**	**0**	**0**	**0**	**1**	**2**	**1**	**0**	**1**	**1**	**1**	**10**	**4**	**9**	**9**	**0**	**3**	**27**	**8**	**0**	**0**	**77**		
**Region B**	**2014**	**1**	**2**	**4**	**0**	**3**	**8**	**0**	**0**	**1**	**0**	**21**	**20**	**27**	**36**	**0**	**2**	**8**	**8**	**4**	**0**	**145**	**17.04**	
	**2015**	**14**	**7**	**3**	**0**	**18**	**4**	**0**	**0**	**0**	**0**	**31**	**20**	**28**	**15**	**2**	**0**	**2**	**0**	**1**	**0**	**145**		
	**2016**	**3**	**4**	**5**	**3**	**6**	**16**	**0**	**0**	**2**	**0**	**102**	**84**	**38**	**56**	**24**	**9**	**6**	**5**	**0**	**0**	**363**		
**Region C**	**2014**	**0**	**6**	**5**	**10**	**4**	**15**	**0**	**0**	**0**	**0**	**80**	**129**	**50**	**60**	**0**	**0**	**20**	**10**	**1**	**0**	**390**	**40.38**	
	**2015**	**0**	**0**	**0**	**1**	**0**	**0**	**0**	**0**	**2**	**0**	**59**	**50**	**102**	**103**	**155**	**146**	**19**	**12**	**4**	**0**	**653**		
	**2016**	**0**	**0**	**1**	**0**	**1**	**0**	**0**	**0**	**2**	**0**	**176**	**132**	**31**	**59**	**40**	**49**	**8**	**4**	**1**	**0**	**504**		
**Region D**	**2014**	**0**	**0**	**3**	**2**	**0**	**2**	**0**	**0**	**0**	**0**	**4**	**5**	**48**	**31**	**47**	**31**	**7**	**3**	**0**	**0**	**183**	**7.83**	
	**2015**	**1**	**0**	**1**	**1**	**0**	**0**	**0**	**0**	**0**	**3**	**10**	**17**	**10**	**29**	**0**	**3**	**6**	**9**	**0**	**0**	**90**		
	**2016**	**0**	**0**	**0**	**0**	**0**	**0**	**0**	**0**	**0**	**0**	**14**	**8**	**2**	**2**	**0**	**0**	**1**	**0**	**0**	**0**	**27**		
**Region E**	**2014**	**0**	**0**	**0**	**0**	**0**	**0**	**0**	**0**	**1**	**0**	**0**	**0**	**5**	**3**	**1**	**2**	**10**	**13**	**0**	**0**	**35**	**0.91**	
**Region F**	**2014**	**0**	**2**	**3**	**1**	**1**	**0**	**0**	**0**	**1**	**1**	**11**	**6**	**3**	**2**	**0**	**0**	**4**	**2**	**0**	**0**	**37**	**0.96**	
**Region G**	**2014**	**3**	**4**	**2**	**2**	**2**	**0**	**0**	**0**	**0**	**0**	**8**	**7**	**13**	**13**	**1**	**1**	**36**	**45**	**0**	**0**	**137**	**3.57**	
**Total (%)**		**59** **(1.54)**		**76** **(1.98)**		**124** **(3.23)**		**1** **(0.02)**		**21** **(0.54)**		**1253** **(32.70)**		**1146** **(29.91)**		**710** **(18.53)**		**426** **(11.11)**		**15** **(0.39)**		**3831**		

F: female, M: male, Nbre: Number, and %: percentage.

*: *P*-value using the non-parametric statistical Kruskal-Wallis method and showing differences between the number of sand fly species in each site.

**: *P*-value using the Chi-square (Fisher’s exact) test and showing the differences between the abundance of sand flies in each region. Region E, F, and G: were excluded from the statistical analysis.

### *Leishmania* detection and typing

**Fig 1:**
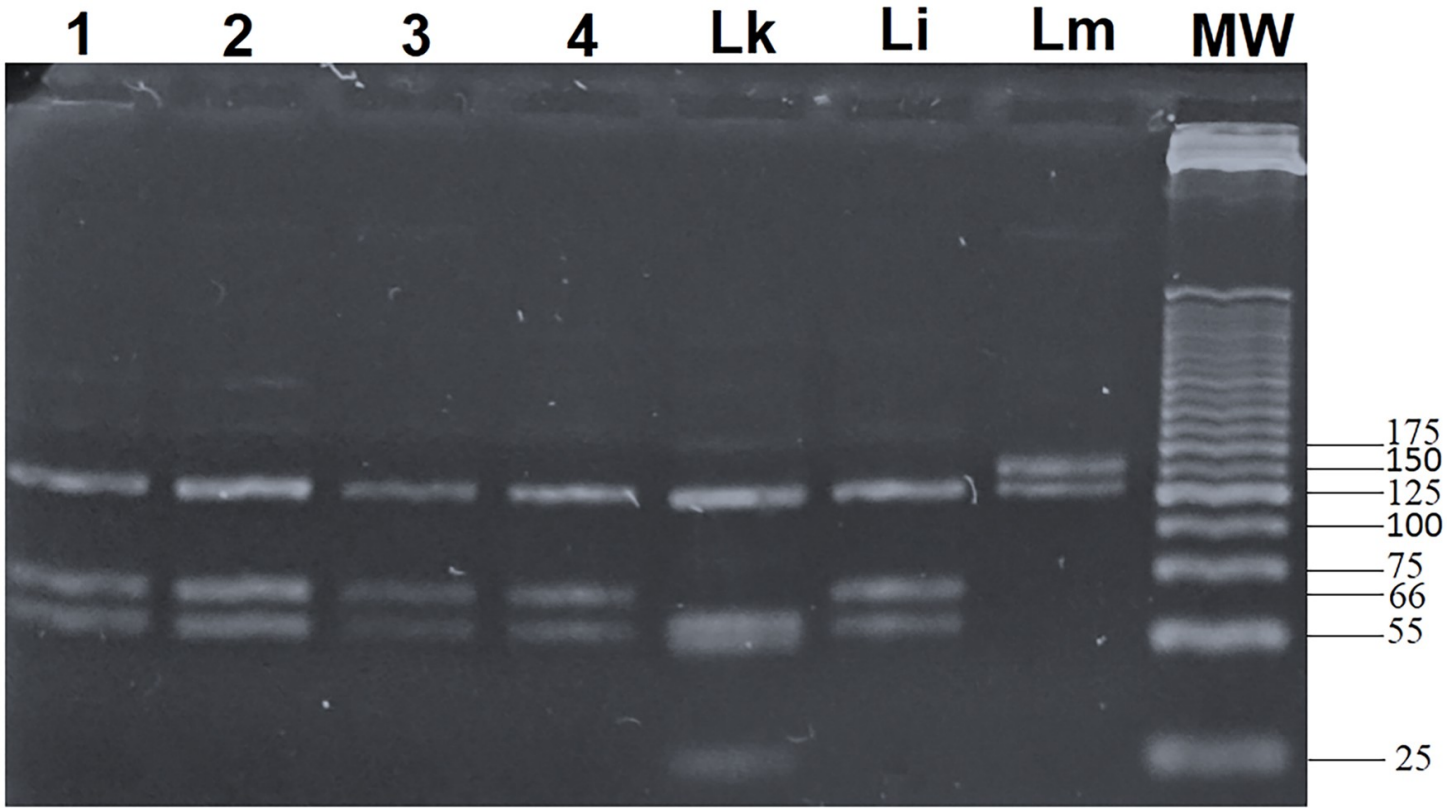
ITS1 nested PCR-RFLP results. The *Leishmania* species typing using RFLP was done for the 54 PCR-ITS1 positive samples. The specific molecular band sizes are: 127, 55, 52, and 20 bp for *L*. *killicki*; 126, 66, and 55 bp for *L*. *infantum*; 145 and 126 bp for *L*. *major*. Lanes 1, 2, and 3: *L*. *infantum* identified from infected *Ph*. *perfiliewi*, Lane 4: *L*. *infantum* identified from infected *Ph*. *perniciosus*, Lk: *L*. *killicki* control, Li: *L*. *infantum* control, and Lm: *L*. *major* control. MW: Molecular Weight marker (25-bp DNA ladder).

Parasite detection was carried out only in *Lar*. *sub*genus females, the suspected vector of *L*. *infantum*. A total of 1,029 females were collected and screened for *Leishmania* infection during the three years of the study. Among this population, 85 specimens were found infected with *Leishmania* spp. (8.26%). *Ph*. *perniciosus* (N = 32; 37.65%) was the most infected species followed by *Ph*. *perfiliewi* (N = 31; 36.47%) and *Ph*. *longicuspis* (N = 17; 20%). For some infected sand flies belonging to *Phlebotomus Lar*. *sub*genus (N = 5; 5.88%), the sand fly species identification was not possible. The one-way ANOVA test was applied to analyze the infection rates among the different sand flies species. Differences were statistically significant (*p*-value = 0.0452). Within the infected sand flies, 62 were unfed (72.94%), 22 were engorged (25.88%), and 1 was gravid (1.17%). Nested PCR for ITS1 was positive in 54 of the cases (63.52%) and negative in 31 cases (36.47%) (*Ph*. *perniciosus*, n = 7, *Ph*. *perfiliewi*, n = 13, *Ph*. *longicuspis*, n = 10 and *Larroussius* unidentifiable sand fly species, n = 1). The typing of *Leishmania* species using RFLP was done for 54 PCR-ITS1 positive samples (Fig 1). Among the infected *Ph*. *perniciosus*, *Leishmania* was identified as *L*. *maj*or (N = 6; 18.75%), *L*. *infantum* (N = 12; 37.50%) and *L*. *killicki* (N = 7; 21.87%). In *Ph*. *perfiliewi* infected sand flies, *Leishmania* was identified as *L*. *infantum* (N = 9; 29.03%), *L*. *major* (N = 7; 22.58%) and *L*. *killicki* (N = 2; 6.45%). The typing of *Leishmania* in *Ph*. *longicuspis*, revealed *L*. *infantum* (N = 4; 23.52%), *L*. *major* (N = 2; 11.76%) and *L*. *killicki* (N = 1; 5.88%). Furthermore, species identification was made for five unidentified *Lar*. species (5.88%) and revealed *L*. *major* (N = 3; 60%), and *L*. *killicki* (N = 1; 20%) (Table 3).

**Table 3:** *Leishmania* typing DNA in infected sand flies.

*Leishmania* typing	*L*. *major*			*L*. *infantum*			*L*. *killicki*			NI *Leishmania*. *spp*.			Total infected sand flies (%)	*P*
Abdomen stage	E	G	UN	E	G	UN	E	G	UN	E	G	UN		
Sand fly species														
***Ph*. *perniciosus***	2	0	4	5	0	7	1	0	6	2	1	4	32 (37.64%)	P* = 0.0452
***Ph*. *perfiliewi***	4	0	3	2	0	7	0	0	2	3	0	10	31 (36.47%)	
***Ph*. *longicuspis***	1	0	1	0	0	4	0	0	1	0	0	10	17 (20.00%)	
**Unidentified** ***Ph*. (*Lar*.) spp.**	2	0	1	0	0	0	0	0	1	0	0	1	5 (5.88%)	
**Total (%)**	9	0	9	7	0	18	1	0	10	5	1	25	85	
	18 (21.17%)			25 (29.41%)			11 (12.94%)			31 (36.47%)				

E: engorged, UN: unfed, G: gravid, and NI: non identified *Leishmania* species. P*: p-value using the one-way ANOVA test.

### *Leishmania* DNA sequencing and phylogenetic analysis

**Fig 2:**
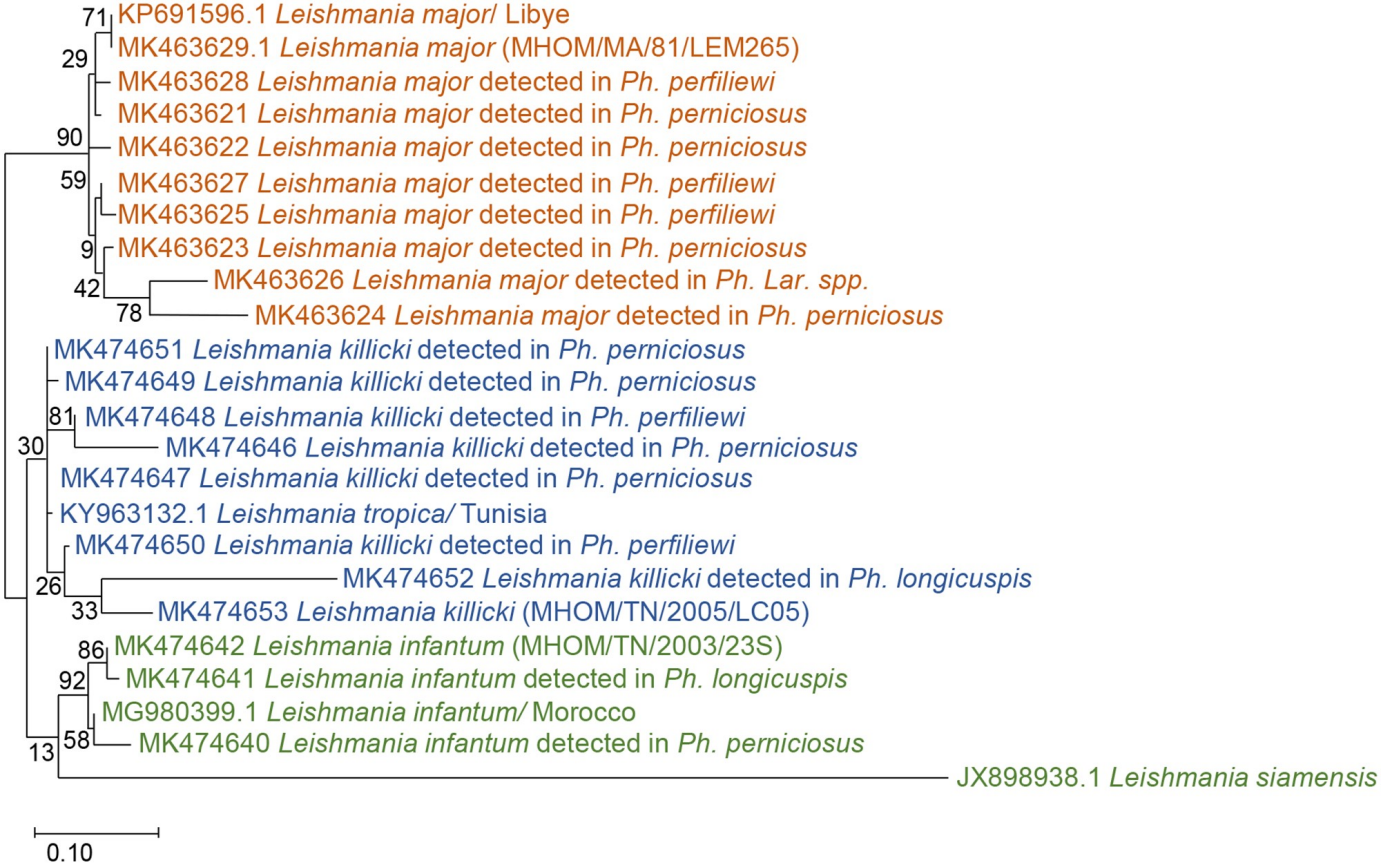
Phylogenetic analysis by Maximum Likelihood method based on *Leishmania* ITS1 sequences. The numbers above the branches indicate the bootstrap sampling percentages.

In order to confirm PCR-RFLP results, amplification products from the ITS1 nested PCR of seventeen infected sand flies and three reference strains of *L*. major (MK463629), *L*. *killicki* (MK474653), and *L*. *infantum* (MK474642) were sequenced. The obtained twenty partial ITS1 DNA sequences were compared to *Leishmania* spp. from GenBank. The phylogenetic analysis was performed to confirm the genetic relationship between strains isolated in human and those detected in sandflies. The ITS1 sequences exhibit a length range of 245–271 bp depending on the species. The topology of the phylogenetic tree showed a clear subdivision in three well-supported clades, corresponding to three *Leishmania* species (*L*. *infantum*, *L*. *killicki*, and *L*. *major*). The identified *L*. *infantum* strains in infected *Ph*. *perniciosus* and *Ph*. *longicuspis* were similar to *L*. *infantum* isolated from human VL (*L*. *infantum* MON-1) from Tunisia (MK474642) and Moroccan strain from human VL (MG980399). *Leishmania major* sequences identified from infected sand flies were clustered with *L*. *major* strain from patients with CL patients from Tunisia (MK463629) and *L*. *major* from Libya (KP691596). *Leishmania killicki* sequences identified in our study were similar to Tunisian *L*. *killicki* strain (MK474653) isolated from human CL and *L*. *tropica* from *Psammomys vexillaris* from Tunisia (KY963132). The phylogenetic analysis based on ITS1 sequences revealed that sequences identified in infected sand flies, sequences from human with CL and VL, and sequence from infected animal were clustered together (Fig 2).

### Blood meal analysis and sequencing

**Fig 3:**
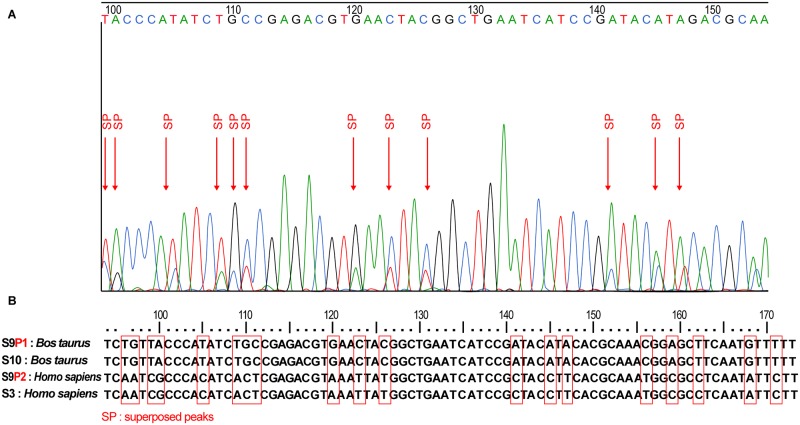
Blood meal identification results. **A:** electropherogram of part of the sequence **S 9** showing superposed peaks, **B:** part of the alignment of four cytochrome *b* sequences showing superposed peaks for S9, **S 10:** cytochrome *b* sequence of *Bos taurus*, **S 3:** cytochrome *b* sequence of *Homo sapiens*, **S 9:** cytochrome *b* sequence of mixed blood meal (*Bos taurus*/*Homo sapiens*), two peaks were detected **peak 1** (**S9P1** corresponding to *Bos taurus* sequence) and **peak 2** (**S9P2** corresponding to *Homo sapiens* sequence).

In order to identify the blood meal sources in the sand flies belonging to *Lar*. *sub*genus, DNA extracted from 177 engorged females sand flies were studied. DNA amplification targeting vertebrate *cyt b* was performed and a 359 bp PCR product was detected in 152 samples (85.87%). Furthermore, negative samples were analyzed using degenerated *cyt b* primers and gave positive results in 17 samples. Thus, a total of 169 positive PCR products (95.48%) were sequenced and compared to the GenBank database. The majority of sand flies were fed on cattle (*Bos taurus*; N = 66; 39.05%), followed by human (*Homo sapiens*; N = 42; 24.85%), sheep (*Ovis aries*; N = 10; 5.91%), chicken (*Gallus gallus*; N = 8; 4.73%), goat (*Capra hircus*; N = 7; 4.14%), donkey (*Equus asinus africanus*; N = 2; 1.18%), and turkey (*Meleagris gallopavo*; N = 1; 0.59%) (Table 4). In addition, the analysis of *cyt b* sequence revealed superposed peaks in the electropherogram at different positions suggesting mixed blood sources (Fig 3). Eight vertebrate host combinations were identified (cow/human; N = 3; 1.77%), (chicken/cow; N = 2; 1.18%), (chicken/sheep; N = 2; 1.18%), (sheep/human; N = 2; 1.18%), (goat/human; N = 1; 0.59%), (sheep/goat; N = 1; 0.59%), (human/rodent; N = 1; 0.59%), and (chicken/turkey; N = 1; 0.59%) (Table 4).

**Table 4:** Blood meal typing in *Ph*. (*Lar*.) species.

Blood meal typing	Cow *Bos taurus*	Human *Homo sapiens*	Sheep *Ovis aries*	Chicken *Gallus gallus*	Goat *Capra hircus*	Donkey *Equus asinus africanus*	Turkey *Meleagris gallopavo*	Human/ Cow *Homo sapiens*/ *Bos taurus*	Chicken/ Cow *Gallus gallus*/ *Bos taurus*	Chicken/ Sheep *Gallus gallus*/ *Ovis aries*	Human/ Sheep *Ovis aries*/ *Homo sapiens*	Goat/ Human *Capra hircus*/ *Homo sapiens*	Chicken/ Guineafowl *Gallus gallus*/ *Agelastes meleagrides*	Sheep/ Goat *Ovis aries*/ *Capra hircus*	Human/ Black rat *Homo sapiens*/ *Volemys musseri*	Not assigned sequences	Total
Sand flies species																	
***Ph*. *perniciosus***	18	19	4	2	6	0	0	0	1	1	2	1	1	1	0	13	69
***Ph*. *perfiliewi***	37	10	4	4	1	0	0	1	1	1	0	0	0	0	1	2	62
***Ph*. *longicuspis***	5	7	2	1	0	1	1	0	0	0	0	0	0	0	0	3	20
**Unidentified *Lar*. spp.**	6	6	0	1	0	1	0	2	0	0	0	0	0	0	0	2	18
**Total (%)**	66 (39.05%)	42 (24.85%)	10 (5.91%)	8 (4.73%)	7 (4.14%)	2 (1.18%)	1 (0.59%)	3 (17.75%)	2 (1.18%)	2 (1.18%)	2 (1.18%)	1 (0.59%)	1 (0.59%)	1 (0.59%)	1 (0.59%)	20 (11.83%)	169

Interestingly, 21 infected sand flies were engorged. The blood meal analysis in infected sand flies revealed a predominance of cattle (N = 11; 52.38%), followed by goat (N = 4; 19.04%), and chicken (N = 2; 9.52%). Even more, we detected mixed blood meals in several specimens: human/cow (N = 1; 4.76%), chicken/cow (N = 1; 4.76%), chicken/sheep (N = 1; 4.76%), and chicken/turkey (N = 1; 4.76%) (Table 5).

**Table 5:** The blood meal origins in infected *Ph*. (*Lar*.) species.

Blood meal origin		Cow *Bos taurus*	Chicken *Gallus gallus*	Goat *Capra hircus*	Chicken/ Cow *Gallus gallus*/ *Bos taurus*	Chicken/ Sheep *Gallus gallus*/ *Ovis aries*	Chicken/ Guineafowl *Gallus gallus*/ *Agelastes meleagrides*	Human/ Cow *Homo sapiens*/ *Bos taurus*	Total
Sand flies and *Leishmania* species									
*Ph*. *perniciosus*	*L*. *major*	1	0	1	0	0	0	0	2
	*L*. *infantum*	1	0	3	0	0	0	1	5
	*L*. *killicki*	0	0	0	1	0	0	0	1
	NI	1	0	0	0	0	1	0	2
*Ph*. *perfiliewi*	*L*. *major*	3	1	0	0	0	0	0	4
	*L*. *infantum*	0	1	0	0	1	0	0	2
	NI	2	0	0	0	0	0	0	2
*Ph*. *longicuspis*	*L*. *major*	1	0	0	0	0	0	0	1
Unidentified *Lar*. spp.	*L*. *major*	2	0	0	0	0	0	0	2
Total (%)		11 (52.38%)	2 (9.52%)	4 (19.04%)	1 (4.76%)	1 (4.76%)	1 (4.76%)	1 (4.76%)	21

NI: non identified *Leishmania* species.

### Parasite load quantification

**Fig 4:**
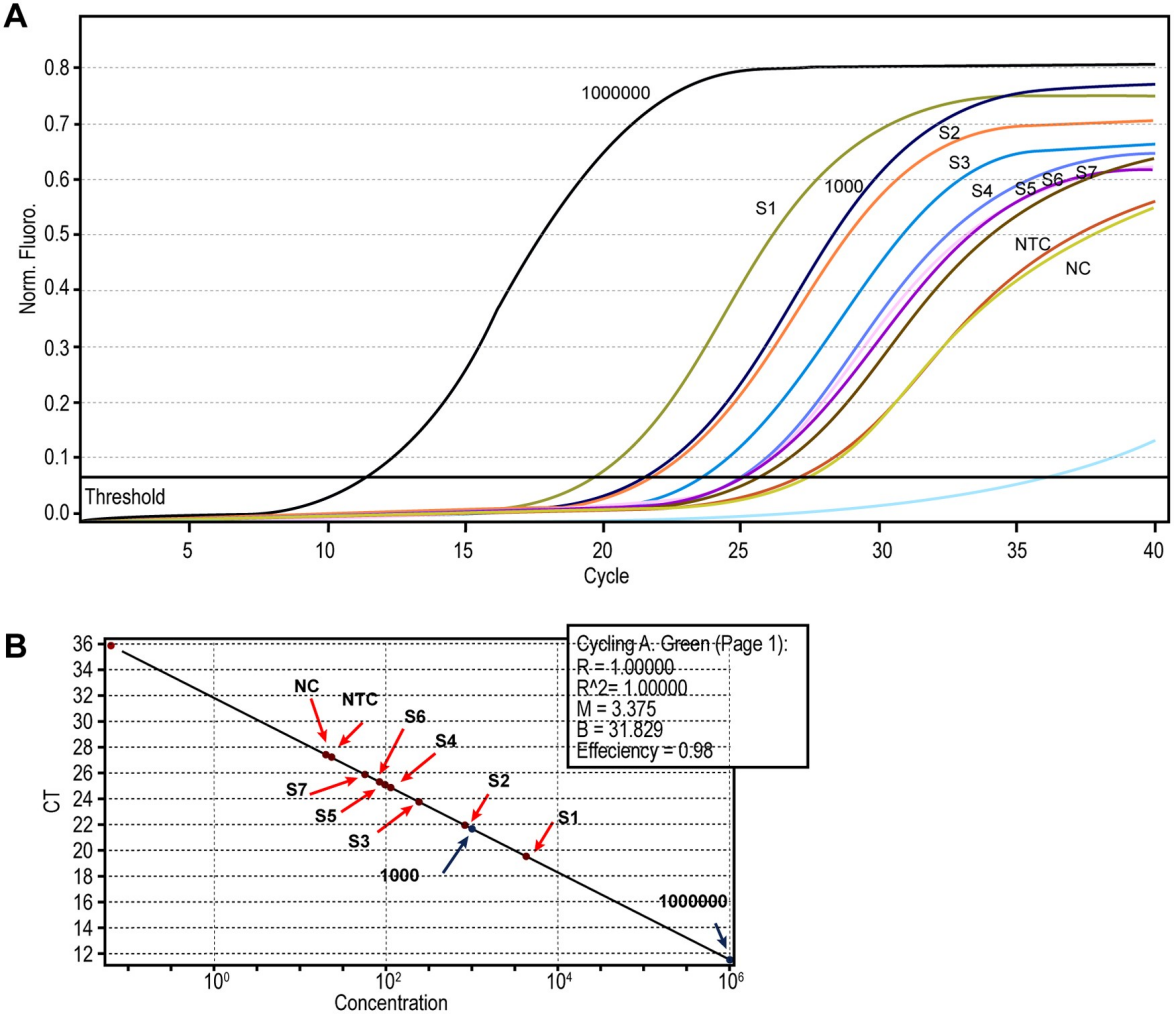
Real time PCR results. **A:** Representative fluorescence acquisition graph showed comparison of each sand fly according to standards. NTC (non template control), NC (negative control corresponding to 30 ng of DNA from reared sand fly), and standards (10^6^ and 10^3^ promastigotes/ml). **B:** Graphic showing concentration results of each sample according to standards. Slope = -3.37, efficiency = 0.98. S1: *Ph*. *perfiliewi* infected with *L*. *infantum*, S2: *Ph*. *perniciosus* infected with *L*. *major*, S3: *Ph*. *perniciosus* infected with *L*. *killicki*, S4: *Ph*. *perfiliewi* infected with *L*. *major*, S5: *Ph*. *(Lar*.*) spp*. infected with *L*. *major*, S6: *Ph*. *perniciosus* infected with *L*. *infantum*, and S7: *Ph*. *perniciosus* infected with *L*. *killicki*.

The parasite load was quantified using qPCR in 17 infected sand flies (6 *Ph*. *perniciosus*, 10 *Ph*. *perfiliewi* and 1 belonging to *Lar*. *sub*genus). Seven of them were engorged, and ten were unfed. Ct threshold values were calculated according to default parameters and NTC Ct values in each reaction (Fig 4). Infected flies were classified in four categories according to the parasite loads: very high loads (≥10,000 parasites/reaction), high loads (≥1,000 parasites/ reaction), moderate loads (> 10 parasites/ reaction), and low parasite loads (<10 parasites/ reaction). The highest parasite load was observed in unfed *Ph*. *perfiliewi* infected with *L*. *infantum* (10,000 parasites) and the lowest was observed in *Ph*. *perfiliewi* infected by *L*. *infantum* (19.9 promastigotes/ reaction) (Table 6). The mean parasite burden in unfed sand flies was 1,174 promastigotes/ reaction, while in engorged females was 90 promastigotes/ reaction. Statistical analysis through Fisher’s exact test showed significant difference between the parasite loads in fed and unfed sand flies (p<0.0001).

**Table 6:** Parasite loads and blood meal analysis in infected sand flies collected in human visceral leishmaniasis focus.

Species	Abdomen states/ blood feed sources	*Leishmania* spp.	Parasite loads estimation (NO. of parasites/ reaction)	Level of infection
***Ph*. *perniciosus***	E/*Capra hircus*	*L*. *major*	100	Moderate
***Ph*. *perfiliewi***	E/ *Bos taurus*	*L*. *major*	100	Moderate
***Ph*. *perfiliewi***	E/ *Bos taurus*	*L*. *major*	199.5	Moderate
**NI** ***Ph*. (*Lar*.) spp.**	E/ *Bos taurus*	*L*. *major*	100	Moderate
***Ph*. *perniciosus***	E/ (*Gallus gallus/Bos taurus*)	*L*. *killicki*	50.1	Moderate
***Ph*. *perfiliewi***	E/ *Gallus gallus*	*L*. *infantum*	19.9	Moderate
***Ph*. *perfiliewi***	E/ (*Gallus gallus/Ovis aries*)	*L*. *infantum*	50.1	Moderate
***Ph*. *perfiliewi***	UN	*L*. *infantum*	10000	Very high
***Ph*. *perniciosus***	UN	*L*. *major*	100	Moderate
***Ph*. *perfiliewi***	UN	*L*. *infantum*	316.2	Moderate
***Ph*. *perfiliewi***	UN	*L*. *infantum*	3981	High
***Ph*. *perfiliewi***	UN	*L*. *infantum*	316.2	Moderate
***Ph*. *perfiliewi***	UN	*L*. *infantum*	1000	High
***Ph*. *perfiliewi***	UN	*L*. *infantum*	50.1	Moderate
***Ph*. *perniciosus***	UN	*L*. *major*	794.3	Moderate
***Ph*. *perniciosus***	UN	*L*. *infantum*	3981	High
***Ph*. *perniciosus***	UN	*L*. *killicki*	199.5	Moderate

E: engorged, UN: unfed, and NI: non identified species.

## Discussion

This study describes the results of an epidemiological study carried out in the center of Tunisia to identify the life cycle of *L*. *infantum* and the potential vectors and reservoirs. The studied region is characterized by a high prevalence of CL and VL and the co-existence of three *L*. *infantum* zymodemes (MON-1, MON-24, and MON-80) [3]. Within the seven monitored sites of human VL, nine sand fly species were identified. *Phlebotomus papatasi* was the predominant (32.70%) followed by *Ph*. *perniciosus* (29.91%), *Ph*. *perfiliewi* (18.35%), *Ph*. *longicuspis* (11.11%), *Ph*. *sergenti* (0.54%), and *Sergentomyia* genus (6.78%). *Phlebotomus papatasi*, the vector of *L*. *major* in Tunisia, is known to be abundant in arid and Saharan regions. Aridity seems to be a limiting factor for its distribution and it was described especially in non-irrigated areas [18, 42–45]. However, the high number of *Ph*. *papatasi* described in our study (32.70%) is in opposition to previously cited studies and it seems that *Ph*. *papatasi* is also predominant in the irrigated area. In the Mediterranean basin, *Ph*. *perniciosus* is described in both humid and arid bioclimatic regions. In Algeria, it was described that the distribution of *Ph*. *perniciosus* spreads out to the Saharan region [46–48]. In Tunisia, *Ph*. *perniciosus* was known to be more frequent in the semi-arid bioclimatic region and much less abundant in sub-humid and arid areas [49, 50]. Recently, in a study conducted in the center of Tunisia, it was demonstrated that *Ph*. *perniciosus* was predominant in arid bioclimatic zones and irrigated areas [42, 51]. Our results corroborate such findings with a predominance of 29.91% for *Ph*. *perniciosus*. Furthermore, *Ph*. *perfiliewi* was known to be limited in the north part of Tunisia and aridity appeared to be a limiting factor for its distribution [49]. Recently, it was demonstrated that *Ph*. *perfiliewi* is the most abundant species in the irrigated area, and its geographical distribution is extending towards the center and the south of Tunisia [18]. Our findings align with the extension of *Ph*. *perfiliewi* (18.53%) towards the center and irrigated areas. Regarding to *Phlebotomus longicuspis*, it was described as no limited to any climatic zone in Tunisia, and appears to have the same distribution in Morocco since it has been found in all biogeographical areas including Saharan regions [51, 52]. In our study, this species was found in semi-arid and irrigated zones (11.11%). Thus, the increase in the trapping of sand flies belonging to *sub*genus *Lar*. (59.95%) is consistent with the hypothesis of the extension of VL in arid areas [18]. Our results highlight the extension of *Lar*. species to the center and their strong involvement in *L*. *infantum* transmission. *Phlebotomus sergenti*, the vector of *L*. *killicki* in Tunisia, was known as the dominant species in the south-east of the country [49]. The low number of *Ph*. *sergenti* observed in our study shows (0.54%) the expansion of this species towards the center. Thus, we demonstrate that the geographical dissemination and sand flies species abundance would be related to several factors associated to human activities and environmental changes (global warming and irrigation) as it was described before [53, 54].

In addition to sand flies species analysis, we screened for *Leishmania* infection in *Lar*. species. The overall rate of infection with *Leishmania* was 8.26%. In agreement with previous reports, our findings show *Ph*. *perniciosus*, *Ph*. *longicuspis*, and *Ph*. *perfiliewi* infected with *L*. *infantum*. Similar results were reported from the central part of Tunisia where *Ph*. *perfiliewi* and *Ph*. *perniciosus* were found infected with *L*. *infantum* [18]. In Algeria, *L*. *infantum* MON-24 was isolated from *Ph*. *perfiliewi*, proving its role in *L*. *infantum* transmission [55]. Such findings highlight the role of *Ph*. *perfiliewi* in *L*. *infantum* transmission in Tunisia and Mediterranean countries. Also, *L*. *infantum* infection was reported in *Ph*. *longicuspis* and *Ph*. *perniciosus* on the eastern coast of Tunisia [19]. Similar results were described in *Ph*. *longicuspis* from Morocco and Algeria [52, 56]. In the light of these findings, *Ph*. *longicuspis* is highly involved in *L*. *infantum* transmission.

Even more, we report for the first time the infection of *Ph*. (*Lar*.) species with *L*. *major* and *L*. *killicki*. Experimental studies described that *Ph*. *perniciosus* is a permissive vector for *L*. *infantum* (MON-1, MON-24, and MON-80), *L*. *major*, and *L*. *tropica* [57, 58]. Furthermore, we describe the incrimination of *Ph*. *longicuspis* and *Ph*. *perfiliewi* in *L*. *major* and *L*. *tropica* transmission.

In addition to molecular detection of *Leishmania*, we carried out a qPCR to quantify the parasite load in infected sand flies. In total, 17 sand flies were analyzed (10 unfed and 7 engorged). The average load was 1,174 parasites/reaction in unfed sand flies and 90 parasites/reaction in engorged sand flies. The highest parasite load was observed in unfed sand flies. Concerning to the unfed females, the highest parasite load detected was 10,000 parasites/reaction in a *Ph*. *perfiliewi* infected by *L*. *infantum*. Similar results were described before with wild caught *Ph*. *perniciosus*, *Lu*. *longipalpis* and *Lu*. *migonei* infected by *L*. *infantum* in Spain and Brazil, respectively [20, 27]. To the best of our knowledge, none of the previous studies has quantified parasite load in *Ph*. *perfiliewi*. Hence, we report the first quantification of *L*. *infantum* in *Ph*. *perfiliewi*. Investigations conducted by Roger et al. (2007) indicated that *Leishmania* parasites could manipulate sand fly feeding behavior. The high parasite loads in sand fly midguts are correlated with a persistent feeding pattern and lead to an increase in *Leishmania* transmission [21]. According to the criteria of Killick-Kendrick (1990), the incrimination of a sand fly species as a vector of leishmaniasis is based on a significant anthropophilic behavior, vectorial capacity, simultaneous presence of vector and disease, and the abundance of the vector [59]. In our study, criteria are already available for anthropophilic behavior, detection of *L*. *infantum* DNA in *Ph*. *perfiliewi* (29%), and the high parasite loads quantified in unfed sand flies demonstrate that *Ph*. *perfiliewi* is a potential vector for *L*. *infantum* in Tunisia. Furthermore, we found a moderate load of infection in *Ph*. *perniciosus* infected with *L*. *major* and *L*. *killicki*, which highlight its role as a permissive vector.

In the current study, the molecular identification of blood sources in engorged sand flies belonging to *sub*genus *Lar*. detected seven sources: cattle, human, sheep, chicken, goat, donkey, and turkey. Compared to similar studies, we report the most extensive range of hosts in mixed *L*. *infantum* focus [19, 30]. Interestingly, we identified mixed blood meals in twelve cases. Our results show that *Lar*. species have no host preferences and are opportunistic feeders while disturbed or challenged with less accessible capillary veins as it was demonstrated before for mosquitoes and phlebotomine sand flies behaviors [60, 61]. Although, dogs have been clearly defined as a proven reservoir of *L*. *infantum* in Tunisia and Mediterranean countries [13, 15, 40, 62, 63], in our study no dog blood was detected in engorged *Ph*. (*Lar*) *sub*genus. The presence of a broad host availability could probably explain this results in the vicinity of the traps confirming the opportunistic behavior of species of this *sub*genus. This finding was also reported in previous studies in Tunisian as well as Spanish leishmaniasis foci [19, 30, 40, 54, 64].

*Leishmania* detection in engorged sand flies showed females fed predominantly on cattle, followed by goat and chicken. Moreover, the analysis of superposed peaks of blood meals in infected sand flies revealed multiple blood origins: human/cow, chicken/cow, chicken/sheep and chicken/turkey. To our knowledge, only dogs were described as the reservoir of *L*. *infantum* MON-1 in Tunisia and a potential reservoir of *L*. *infantum* MON-24 and MON-80. In Mediterranean countries, the dog is the main reservoir of VL. However, many other hosts were suspected such as rabbit, cat, hare, jackal, fox, wild rodents, and horse [65–76]. This demonstrates the involvement of different mammals in *L*. *infantum* transmission. Moreover, our results of parasite typing and quantification in engorged sand flies showed an average load of 90 parasites/reaction. Our findings align with previous studies, which found that the highest parasite loads were observed in unfed sand flies [20, 77]. In addition, a moderate load was described for *Ph*. *perfiliewi* and *Ph*. *perniciosus* infected with *L*. *major* and fed on cow and goat, respectively. These findings could be explained by two hypotheses. The first suggests that cow and goat could be potential reservoirs, and the second hypothesis suggests that *Ph*. *perfiliewi* and *Ph*. *perniciosus* could be potential vectors of *L*. *major*. To the best of our knowledge, only rodents have been described as reservoirs of *L*. *major* in Tunisia [14]. Hence, the hypothesis that cow and goat could be a reservoir of *L*. *major* should be discarded. To conclude, our findings support the hypothesis that *Ph*. *perfiliewi* and *Ph*. *perniciosus* could be permissive vectors and the blood was only necessary for eggs maturation.

Unexpectedly, during the blood meal analysis of engorged infected sand flies, we identified mixed blood meals in two *Ph*. *perniciosus* and *Ph*. *perfiliewi* infected with *L*. *killicki* and *L*. *infantum*, respectively. The quantification of the parasite load revealed 50 parasites/reaction in both of them. It was described before using experimental infections that both *L*. *infantum* and *Leishmania mexicana* promote feeding on multiple hosts. Furthermore, it was demonstrated that *Leishmania* parasites could manipulate sand fly feeding behavior according to the presence of infective forms available for transmission [21]. Furthermore, it was recently proved that ingestion of a second blood by *Leishmania* infected sand flies triggers parasite dedifferentiation and amplification that greatly enhance disease transmission [78]. In the light of these findings, the moderate number of parasites observed in mixed blood meals could be explained by the necessity of a second blood meal for dedifferentiation and amplification of parasite as proven previously. In another hand, we observed a low parasite charge (19 parasites/reaction) in *Ph*. *perfiliewi* infected by *L*. *infantum* and fed on chicken. Indeed, it was previously described that chickens or birds are not susceptible to *Leishmania* infection due to some physiological characteristics such as their body temperature of 41°C and infected sand fly may eliminate *Leishmania* parasite during their second blood meal [79].

In light of this finding, the moderate and the low amount of parasite observed in our study demonstrate that chicken blood affects the presence of parasites in the sand fly gut and 50 parasites/reaction could be the quantity of parasite uptaken from the first host. So, the supposition that chicken could be a reservoir for *L*. *infantum* should be discarded. Further studies should be carried out to confirm the involvement of the described vertebrate in *L*. *infantum* life cycle such as experimental infection.

## Conclusion

In the present study, we demonstrated the high involvement of *Ph*. *longicuspis*, *Ph*. *perfiliewi*, and *Ph*. *perniciosus* in *L*. *infantum* transmission. Moreover, we confirmed that *Ph*. *perniciosus* is a permissive vector in nature which strengthens its role in different *Leishmania* life cycles. Thereby, an efficient control strategy against these species and its distribution toward cutaneous and visceral leishmaniasis foci should be carried out in order to reduce *Leishmania* transmission in Tunisia. Moreover, *Ph*. *perfiliewi* could be a permissive vector for *L*. *infantum* and other *Leishmania* species. Thus, it would be preferable to combine sand fly dissection and *Leishmania* strain isolation from *Ph*. *perfiliewi* to determine the *L*. *infantum* zymodeme. The combination of the parasite detection and the blood meal analysis in infected flies revealed the possible incrimination of different mammals in *L*. *infantum* transmission. Such results await further exploration to a better understanding of *L*. *infantum* transmission.

## Supporting information

S1 File Alignment of analyzed *Leishmania* ITS1 sequences used in the phylogenetic analysis. (PDF)
