# Supplementary material for: Molecular detection and identification of Leishmania DNA and blood meal analysis in Phlebotomus (Larroussius) species
Source: PLoS Negl Trop Dis. 2020 Mar 26;14(3):e0008077. doi: 10.1371/journal.pntd.0008077 (PMC7098558; doi:10.1371/journal.pntd.0008077)
Supplement: S1 File — (PDF) [file pntd.0008077.s001.pdf]

```
                   10        20        30        40        50        60        70
          ....|....|....|....|....|....|....|....|....|....|....|....|....|....|
KP691596  GGA-GGCTTATTCTATATATATATATAG---TATAG--GCTT-TTCC-CACATACACAGCAAACTTTTAT
MK463629  GGA-GGCTTATTCTATATATATATATAG---TATAG--GCTT-TTCC-CACATACACAGCAAACTTTTAT
MK463628  GGAAGGCTTATTCTATATATATATATAG---TATAGC-GCTT-TTCC-CACATACACAGCACACTTTTGT
MK463621  GGAAGGCTTATTCTATATATATATATAG---TATAGC-GCTT-TTCC-CACATACACAGCACACTTTTAT
MK463622  GGGAGGCTTATTCTATATATATATATAG---TATAGC-GCTT-TTCC-CACATACACAGCACACTTTTAT
MK463627  AGGAGGCTTATTCTATATATATATATAG---TATAGC-GCTT-TTCC-CACATACACAGCACACTTTTAT
MK463625  AGGAGGCTTATTCTATATATATATATAG---TATAGC-GCTT-TTCC-CACATACACAGCACACTTTTAT
MK463623  GGGAGGCTTATTCTATATATATATATAG---TATGG--GCTT-TTCC-CACATACACAGCACACTTTTAT
MK463626  GAGAGCCTAAACTTCTATATATATATATCCGTATAGC-GCTTATTCC-CACATACACAGCACACTTTTAT
MK463624  GGGAGAGCTATTTTCTATATATATATAGCCTT-TCCC-GCTT-TTCC-CACATACACATCAAATTTTTAT
MK474651  GGGAGGCCTA---TATATAT-TATACATT-ATATAG--GCCTTTCCCACACATACACAGCAAACTTTTGT
MK474649  GGGAGGCCTA---TATATAT-TATACATT-ATATAGA-GCCTTTCCCACACATACACAGCAAACTTTTGT
MK474648  GGAAGGCCTA---TATATATATATACATT-ATATAGG-GCCTTTCCCACACATACACAGCAAACTTTTGT
MK474646  AGAAGGCCTA---TATATAT-TATACAGT-ATATAC--GCCTTCCCCACACATACACAGCAAACTTTTGT
MK474647  GGGAGGCCTA---TATATAT-TATACATT-ATATAG--GCCTTTCCCACACATACACAGCAAACTTTTGT
KY963132  GGGAGGCCTA---TATATAT-TATACATT-ATATAG--GCCTTTCCCACACATACACAGCAAACTTTTGT
MK474650  GGGAAGCCTA---TATATAT-TATACATT-ATATAG--GCCTTTCCCACACATACACAGCAAACTTTTGT
MK474652  AGGATGCCTA---TATATAT-TATACGTT-TTACCG--GCTTTTCCCCCCCTTACCCGTCAACCTTTTTT
MK474653  AGGAAGCCTA---TATATAT-TATACATT-TTATATAGGCCTTTCCCACACATACACAGCAAACTTTTGT
MK474642  GGGGAGACTA---TGTATATATATGTAG----------GCCTTTCCC--ACATACACAGCAAAGTTTTGT
MK474641  AAGGAGACTA---TGTATATATATGTAG----------GCCTTTCCC--ACATACACAGCAAAGTTTTGT
MG980399  GGGAGACCTA---TGTATATATATGTAG----------GCCTTTCCC--ACATACACAGCAAAGTTTTGT
MK474640  AGAAGACCTAA--TGTATATATATGTAG----------GCCTTTCCC--ACATACACAGCAAAGTTTTGT
JX898938  TAGAGAGTAGTAGAATACATCTACTCGGGGAGGCAT--GTTTTTTCC-----GATATGCCTTTCCCACAT

                   80        90       100       110       120       130       140
          ....|....|....|....|....|....|....|....|....|....|....|....|....|....|
KP691596  ACTCGAAATTTGCAGTAAA-----AAAGGCCGATCGACGTTGTAGAACGCACCGCCTATAC--ACAAAAG
MK463629  ACTCGAAATTTGCAGTAAA-----AAAGGCCGATCGACGTTGTAGAACGCACCGCCTATAC--ACAAAAG
MK463628  ACTCGAAATTTGCAGTAAA-----AAAGGCCGATCGACGTTGTAGAACGCACCGCCTATAC--ACAAAAG
MK463621  ACTCGAAATTTGCAGTAAA-----AAGGGCCGATCGACGTTGTAGAACGCACCGCCTATAC--ACAAAAG
MK463622  ACTCGAAATTTGCAGTAAA-----AAAGGCCGATCGACGTTGTAGAACGCACCGCCTATAC--ACAAAAG
MK463627  ACTCGAAATTTGCAGTAAA-----AAGGGCCGATCGACGTTGTAGAACGCACCGCCTATAC--ACAAAAG
MK463625  ACTCGAAATTTGCAGTAAA-----AARGGCCGATCGACGTTGTAGAACGCACCGCCTATAC--ACAAAAG
MK463623  ACTCGATATTTGCAGTAAA-----AAGGGCCGATCGACGTTGTAGAACGCACCGCCTATAC--ACAAAAG
MK463626  ACTCGATATTTGCAGTAAA-----AAGCGCCGATCGACGTTGTAGAACGCACCGCCTATAC--ACAAAAG
MK463624  TCTCAATATTTGCAGGAAA-----AAG-GCCGATCGACGTTGTAGAACGCACCGCCCATAC--ACAAAAG
MK474651  ACTCGAAGTTTGCAGTAAACA---AAAGGCCGATCGACGTTATA--ACGCACCGCCTATAC--ACAAAAG
MK474649  ACTCGAAGTTTGCAGTAAACA---AAAGGCCGATCGACGTTATA--ACGCACCGCCTATAC--ACAAAAG
MK474648  ACTCGAAGTTTGCAGTAAACA---AAAGGCCGATCGACGTTATA--ACGCACCGCCTATAC--ACAAAAG
MK474646  ACTCTAAGTTTGCAGTAAACA---AAAGGCCTATCGACGTTATA--ACGCACCGCCTATAC--ACAAAAT
MK474647  ACTCGAAGTTTGCAGTAAACA---AAAGGCCGATCGACGTTATA--ACGCACCGCCTATAC--ACAAAAG
KY963132  ACTCGAAGTTTGCAGTAAACA---AAAGGCCGATCGACGTTATA--ACGCACCGCCTATAC--ACAAAAG
MK474650  ACTCGAAGTTTGCAGTAAACA---AAAGGCCGATCGACGTTATA--ACGCACCGCCTATAC--ACAAAAG
MK474652  ACTCAAAATTTGCGCTAAACA---AAAGGCCAACCCACCTTATA--ACCCACCGCCTTTCC--ACAAAAT
MK474653  ACTCGAAGTTTGCAGTAAACA---AAAGGCCAATCAACGTTATA--ACCCACCCCCAATAC--ACAAAAC
MK474642  ACTCAAAATTTGCAGTAAAAA---AAAGGCCGATCGACGTTATA--ACGCACCGCCTAT----ACAAAAG
MK474641  ACTCAAAATTTGCAGTAAAAA---AAAGGCCGATCGACGTTATA--ACGCACCGCCTAT----ACAAAAG
MG980399  ACTCAAAATTTGCAGTAAAAA---AAAGGCCGATCGACGTTATA--ACGCACCGCCTAT----ACAAAAG
MK474640  ACTCAAAATTTGCAGTAAAAA---AAAGGCCGATCGACGTTATA--ACGCACCGCCTAT----ACAAAAG
JX898938  ACACAAACACAGCAATATATATGTATATATATACGTATATTGCTATACCCAAAAACCATACCGTAAAAAG

                  150       160       170       180       190       200       210
          ....|....|....|....|....|....|....|....|....|....|....|....|....|....|
KP691596  CAAAAATGTCCGTTTATACAAAA------AAATAGACGGCGTTTCGGTT--TTTGGCGGGAGGGAGAGAG
MK463629  CAAAAATGTCCGTTTATACAAAA------AAATAGACGGCGTTTCGGTT--TTTGGCGGGAGGGAGAGAG
MK463628  CAAAAATGTCCGTTTATACAAAA------AAATAGACGGCGTTTCGGTT--TTTGGCGGGAGGGAGAGAG
MK463621  CAAAAATGTCCGTTTATACAAAA------AAATAGACGGCGTTTCGGTT--TTTGGCGGGAGGGAGAGAG
MK463622  CAAAAATGTCCGTTTATACAAAA------AAATAGACGGCGTTTCGGTT--TTTGGCGGGAGGGAGAGAG
MK463627  CAAAAATGTCCGTTTATACAAAA------AAATAGACGGCGTTTCGGTT--TTTGGCGGGAGGGAGAGAG
```

```
MK463625   CAAAAATGTCCGTTTATACAAAA------AAATAGACGGCGTTTCGGTT--TTTGGCGGGAGGGAGAGAG
MK463623   CAAAAATGTCCGTTTATACAAAA------AAATAGACGGCGTTTCGGTT--TTTGGCGGGGGGGAGAGAG
MK463626   CAAAAATGTCCGTTTATACAAAA------ATATAGACTGCGTTTCGGTT--TTTGGCGGGAGGGAGAGAG
MK463624   CAAAATTGTCCGTTTATACAAAA------ATATAGACTGTGTTTCGGTT--TTTGGCGGGGGGGAGAGAG
MK474651   CAAAAATGTCCGTTTATACAAA----------TATACGGCGTTTCGGTT--TT------------GTTGG
MK474649   CAAAAATGTCCGTTTATACAAA----------TATACAGCGTTTCGGTT--TT------------GTTGG
MK474648   CAAAAATGTCCGTTTATACAAA----------TATACTGCTTTTCGGTT--TT------------GTTGG
MK474646   CTAAAATGTCCGTTTATACAAA----------TATACTGCTTTTCGGTT--TT------------GGTGG
MK474647   CAAAAATGTCCGTTTATACAAA----------TATACGGCGTTTCGGTT--TT------------GTTGG
KY963132   CAAAAATGTCCGTTTATACAAA----------TATACGGCGTTTCGGTT--TT------------GTTGG
MK474650   CAAACATGTCCGTTTATACAAA----------TATACGGCGTTTCGGTT--TT------------GTTGG
MK474652   CAAACATGTCCTTTTATACAAT----------TATACGGCTTTCCGGTT--TT------------GTTGG
MK474653   CAAACATGTCCGTTTATACAAA----------TATACGGCGTTTCGGTT--TT------------GTTGG
MK474642   CAAAAATGTCCGTTTATACAAAA------A-ATATACGGCGTTTCGGTT--TT--------------TGG
MK474641   CAAAAATGTCCGTTTATACAAAA------A-ATATACGGCGTTTCGGTT--TT--------------TGG
MG980399   CAAAAATGTCCGTTTATACAAAA------A-ATATACGGCGTTTCGGTT--TT--------------TGG
MK474640   CAAAAATGTCCGTTTATACAAAA------A-ATATACGGCGTTTCAGTT--TT--------------TGG
JX898938   CAAAAAGGCCGGTCGACGCCAAATGCCGCGCGTATACAGTGGAAAAGTCCGTTCGTTACGGCTCTTTCTC

                   220       230       240       250
           ....|....|....|....|....|....|....|....|....|...
KP691596   AGGGGGGTGCGTGCGCGT-GGATAACGGCTCA-CATAA-CGTGTCGCG
MK463629   AGGGGGGTGCGTGCGCGT-GGATAACGGCTCA-CAT------------
MK463628   AGGGGGGTGCGTGCGCGT-GGATAACGGCTCA-CATAAACGTGTCAGC
MK463621   AGGGGGGTGCGTGCGCGT-GGATAACGGCTCA--ATAAACGTGTCACA
MK463622   AGGGGGGTGCGTGCGCGT-GGATAACGGCTCA-ATAAACCGTGTCACA
MK463627   AGGGGGGTGCGTGCGCGT-GGATAACGGCTCA-CATAAACGTGTCACA
MK463625   AGGGGGGTGCGTGCGCGT-GGATAACGGCTCA--CATAACGTGTCACA
MK463623   AGGGGGGTGCGTGCGCGT-GGATAACGGCTCA--ATAA-CGTGTCACA
MK463626   AGGGGGGGGCGTGCGCGTAGGATAACGGCTCACATAAACGGTGTCACA
MK463624   AGGGGGGG-CGTGC-------ATAACGGCTCA----AAGCATGTCACA
MK474651   CGGGGGGTGCGTGTGTGT-GGATAACGGCTCA-CATAAACGTGTCGCA
MK474649   CGGGGGGTGCGTGTGTGT-GGATAACGGCTCA--ATAAACGTGTCACA
MK474648   CGGGGGGTGCATGTGTGT-GGATAACGGCTCA-CATAAACGTGTCGCA
MK474646   CGGGGGGTGCATGTGTGT-GGACGCA----------------------
MK474647   CGGGGGGTGCGTGTGTGT-GGATAACGGCTCA-CATAA-CGTGTCGCA
KY963132   CGGGGGGTGCGTGTGTGT-GGATAACGGCTCA-CATAA-CGTGTCGCG
MK474650   TGGGGGGTGCGTATGTGT-GGATAACGGCTCA-CATAA-CGTGTCGCA
MK474652   TGGGGGGAGCATGGGTGT-GAATAACGGCTCC-AATAA-CGTGCCCAA
MK474653   GGGGGGGTGCGTGTGTGT-GGATAACGGCTCA-TTAAAC---------
MK474642   CGGGGTGGGTGCGTGTGT-GGATAACGGCTCA-CATAA-CGTGTCGCA
MK474641   CGGGGTGGGTGCGTGTGT-GGATAACGGCTCA-CATAA-CGTGTCGCA
MG980399   CGGGGTGGGTGCGTGTGT-GGATAACGGCTCA-CATAA-CGTGTCGCA
MK474640   CGGGGTGGGTGCGTGTGT-GGATAACGGCTCACATAAAACGTGTCGCA
JX898938   TCTCGCGGGTGTGTGTGT-GGATAACGGCTCA-CATAA-CGTGTCGCG
```
